# Correlates of recent nonfatal overdose among people who inject drugs in West Virginia

**DOI:** 10.1186/s12954-021-00470-y

**Published:** 2021-02-18

**Authors:** N. Jia Ahmad, Sean T. Allen, Rebecca Hamilton White, Kristin E. Schneider, Allison O’Rourke, Michelle Perdue, Charles Babcock, Michael E. Kilkenny, Susan G. Sherman

**Affiliations:** 1 Department of Health Policy and Management, Johns Hopkins University Bloomberg School of Public Health, 624 N. Broadway, Baltimore, MD 21205 USA; 2 Department of Health, Behavior and Society, Johns Hopkins University Bloomberg School of Public Health, 624 N. Broadway, Baltimore, MD 21205 USA; 3 Department of Mental Health, Johns Hopkins University Bloomberg School of Public Health, 624 N. Broadway, Baltimore, MD 21205 USA; 4 DC Center for AIDS Research, Department of Psychological and Brain Sciences, George Washington University, 2125 G St. NW, Washington, DC 20052 USA; 5 Cabell Huntington Health Department, 703 7th Ave., Huntington, WV 25701 USA; 6 Marshall University School of Pharmacy, 1538 Charleston Ave., Huntington, WV 25701 USA

**Keywords:** Injection drug use, Overdose, Rural drug use

## Abstract

**Aim:**

Experiencing a nonfatal overdose (NFOD) is a significant risk factor for a subsequent nonfatal or fatal overdose. Overdose mortality rates in rural Appalachian states are some of the highest in the USA, but little is known about correlates of overdose among rural populations of people who inject drugs (PWID). Our study aimed to identify correlates of experiencing a recent (past 6 months) NFOD among rural PWID in Cabell County, West Virginia.

**Methods:**

Using data from a June–July 2018 cross-sectional survey that was designed to estimate the size and characteristics of the PWID population in Cabell County, West Virginia, we used log binomial regression to identify correlates (e.g., structural vulnerabilities and substance use) of NFOD in the past 6 months.

**Results:**

The majority of our sample of 420 PWID were male (61.2%), White, non-Hispanic (83.6%), and reported recent heroin injection (81.0%). More than two-fifths (42.6%) experienced a recent NFOD. Independent correlates of NFOD included witnessing an overdose in the past 6 months (adjusted prevalence ratio [aPR] = 2.28; 95% CI 1.48–3.50), attempting to quit using drugs in the past 6 months (aPR = 1.54; 95% CI 1.11–2.14), and the number of drugs injected (aPR = 1.16; 95% CI 1.10–1.23)

**Conclusions:**

A large proportion of rural PWID in Appalachia reported having recently overdosed. The associations between witnessing an overdose, attempting to quit using drugs, and number of drugs injected with recent nonfatal overdose underscore the need for expanded access to overdose prevention resources that are tailored to the needs of this population. Expanding access to evidence-based overdose prevention strategies—such as take-home naloxone programs, treatment with methadone or buprenorphine, and harm reduction services—may decrease overdose morbidity and mortality among rural PWID in Appalachia.

## Introduction

In 2017, there were an estimated 109,500 people who died of an opioid-related overdose globally, 43% of whom were in the USA [1]. The opioid crisis has had a devastating impact on the health of Americans, decreasing life expectancy in the USA for three consecutive years [2]. Rural Appalachia is one of the regions that has been most severely impacted by the modern opioid crisis [3]. In West Virginia (WV), the opioid overdose death rate increased 12% annually between 2008 and 2016, and the state has had the highest age-adjusted opioid mortality rate in the USA for the past five consecutive reported years [4, 5].

There are more than 15 million people who inject drugs (PWID) globally [6]. A recent meta-analysis estimated that one-fifth of this population experienced an overdose in the past year [7]. Nonfatal overdose (NFOD) is an important public health concern, not only because of its high prevalence among this vulnerable population, but because it is associated with an increased risk of future overdose mortality and independent harms [8]. NFOD is much more common than fatal overdose and carries significant consequences, including hypoxic brain injury, cardiac and renal problems, and acute traumatic injuries [9–11]. Some studies show that up to 80% of people who have overdosed experience one of the sequelae above [12]. NFOD is also associated with increased healthcare costs [13]. Among people who use drugs, PWID are more physiologically vulnerable to overdose than persons who use drugs via other routes of administration [7]. Injection drug use has a more rapid onset of action than other modalities and can be more challenging to titrate. PWID are also highly vulnerable to fatal and nonfatal overdose as they may knowingly or unknowingly use drugs containing highly potent synthetic opioids, like fentanyl [14, 15].

Research has found that polysubstance use, either intentional concurrent use of multiple drugs or unintentional via adulterants, is a common risk factor for NFOD [7, 9, 16, 17]. Following periods of abstinence from substance use, such as incarceration or attempts to quit without medications for opioid use disorder, biological tolerance for opioids falls, leaving PWID more susceptible to overdose if they resume drug use [18]. The environments in which drug use occurs also affect risk; recent studies conducted among urban PWID in North America suggest that persons who predominately use drugs in public venues are more vulnerable to NFOD [19, 20]. Additional factors associated with increased risk of NFOD include structural vulnerabilities, such as homelessness [7, 21].

The existing overdose literature primarily reflects studies conducted in metropolitan areas [8, 21–23]. The limited literature on correlates of NFOD among rural populations indicates that prescription opioid injection and psychiatric disorders contribute to overdose risks [24]. Previous studies in the USA have also demonstrated that access to naloxone, syringe services programs, and medications for opioid use disorder (MOUD) in rural communities often lags behind urban counterparts [25–30]. There is an ongoing need for enhanced understanding of NFOD in rural communities given the sustained nature of the opioid crisis and associated epidemic of overdose fatalities in non-urban areas. The purpose of this research is to identify correlates of NFOD among a PWID population in a rural county in Appalachia (Cabell County, WV).

## Methods

### Study description

Data are from a June–July 2018 population estimation study among PWID in Cabell County, WV. The study procedures are described elsewhere [31]. Briefly, the study aimed to describe the size, characteristics, and experiences of the PWID population in Cabell County via the capture–recapture method for population size estimation. The capture–recapture method has been used to understand the size and characteristics of marginalized populations, such as PWID and female sex workers [32–36]. This method involves two rounds of survey data collection (i.e., the capture and recapture phases). Participant counts from both phases of data collection (and the overlap between them) can then be used to estimate the size of the target population [31, 37, 38]. In our study, the capture phase of data collection occurred at the syringe services program at the Cabell-Huntington Health Department. The recapture phase of data collection occurred in community locations throughout Cabell County that were known for drug use activities. Locations for participant recruitment in the recapture phase were identified through discussions with local stakeholders (e.g., persons in recovery, staff at the collaborating syringe services program, and persons involved in overdose response). In addition, we conducted geospatial analyses of possible indicators of drug use activities to glean an enhanced understanding of possible community recruitment locations. Eligibility criteria were broad: (1) to be at least 18 years old and (2) to have ever used drugs by any route of administration. Study data were collected anonymously via audio computer-assisted self-interview (ACASI) and participants received incentives ($10 grocery gift card or snack bag) for participation. Our analytic sample for this analysis reflects n = 420 surveys that were completed by persons who indicated they had injected drugs in the past 6 months. The Johns Hopkins Bloomberg School of Public Health Institutional Review Board approved this research.

### Measures

#### Overdose history

We captured lifetime overdose experiences by asking: “Have you ever overdosed to the point of passing out?” If participants answered “yes,” they were asked: “In the past 6 months, how many times have you overdosed to the point of passing out?” Answers to this question were dichotomized to a variable that indicated if persons had overdosed at least once in the past 6 months versus those who had not overdosed in the past 6 months (inclusive of persons who had never overdosed).

#### Sociodemographic measures

Participants reported their age at time of survey completion as a continuous measure. For race, participants were instructed to select all applicable options from the following: White, Black or African American, Asian, Native American or other Pacific Islander, American Indian or Alaska Native, Multiracial, and other. Persons were also asked to report if they identified as Hispanic. Our sample consisted primarily of persons who identified as White and non-Hispanic. As a result, we combined the race and ethnicity measures to a single variable that indicated if persons identified as White, non-Hispanic versus all others. The lack of racial and ethnic diversity in our sample is comparable to the demographic profile of the study setting (the US Census Bureau estimates that 90.1% of Cabell County residents identified as “White alone, not Hispanic/Latino”) [39]. We created a binary measure for relationship status that indicated if persons were single versus in a relationship or married. We included a binary variable indicating if persons had completed high school or equivalent.

#### Structural vulnerability measures

Participants indicated if they considered themselves homeless and currently had health insurance coverage. Participants were also asked about their employment status and food insecurity (defined as going to bed hungry at least once per week versus less than once per week). We also included a binary (yes/no) measure of recent arrest, “Have you been arrested in the past 6 months?”

#### Substance use-related measures

Participants reported substances used in the past 6 months by administration route. Injection drugs included cocaine, crystal methamphetamine, heroin, prescription opioids (e.g., Oxycontin, Percocet, and Codeine), fentanyl, buprenorphine or Suboxone, and speedball (cocaine and heroin). We used these data to create a measure ranging from 1 to 7 to reflect the number of drugs recently injected. Drugs used by other routes of administration included cocaine, crystal methamphetamine, heroin, prescription opioids, fentanyl, buprenorphine or Suboxone, sedatives, tranquilizers, stimulants, and hallucinogens. We collapsed non-injection drug use by drug type (e.g., smoking and snorting a given drug were combined to a single measure of non-injection use of the drug). We also created a dichotomous measure that indicated where persons typically used drugs: private (at your home or at someone else’s home) versus public (on the street; at a park; a stairwell in a building or business; an abandoned building; a public bathroom, at a restaurant; on a bus or train; in a car, truck, or other vehicle; in the woods) locations [19]. Participants who selected “other” location (*n* = 12) or refused to answer (*n* = 1) were recoded as missing.

We also asked if persons typically used drugs alone versus with one or more persons. Participants indicated (yes/no) if they tried to quit using drugs in the past 6 months. Finally, we asked participants how many times in the past 6 months they witnessed a fatal overdose and how many times they witnessed a nonfatal overdose. These responses were recoded to a single binary variable comparing witnessing no overdoses (fatal or nonfatal) in the past 6 months to witnessing at least one (fatal or nonfatal) overdose in the past 6 months; *n* = 2 participants refused to answer both questions and were recoded to missing.

### Statistical analysis

Initial tests of association with experiencing NFOD were calculated using Pearson’s chi-square tests and independent samples t-tests. We used log binomial regression to identify correlates of experiencing NFOD because the prevalence of recent NFOD was high (42.6%). All variables with a p-value < 0.10 in bivariate analyses were considered for inclusion in the multivariable analysis. Multiple correlations were identified among our measures of structural vulnerability and substance use; as a result, we selected those variables for the final model that maximized model fit. Although we collected information about non-injection drug use, our analysis focuses on the number of drugs injected as injection drug use conveys a higher risk of overdose [40]. AIC was used to compare models, and the Hosmer–Lemeshow test was used to assess goodness of fit. We used Stata Version 15 for all analyses.

## Results

The majority of our sample of rural PWID identified as White, non-Hispanic (83.6%), and male (61.2%) (Table 1). Multiple structural vulnerabilities were prevalent in our sample. Most participants reported being homeless (56.0%), unemployed (66.0%), and experiencing food insecurity (64.8%). Heroin was the most commonly (81.0%) reported recent (past 6 months) injection drug. Most participants also indicated that they used drugs with others (68.3%), witnessed at least one overdose in the past 6 months (73.0%) and attempted to quit using drugs in the past 6 months (74.5%). More than two out of five (42.6%) experienced an overdose in the past 6 months and, among this group, the average number of recent personal overdoses was 5.2 (SD 8.2).

**Table 1:** Sociodemographics, structural vulnerabilities, and substance use measures among people who inject drugs in Cabell County, WV 2018 (n = 420)

	Total	Did not overdose in past 6 months (n = 241, 57.4%)	Overdosed at least once in past 6 months (n = 179, 42.6%)	*P* value
*Sociodemographics*				
Age (mean, SD)	35.8 (8.5)	35.8 (8.7)	35.7 (8.4)	0.855
Male	257 (61.2)	149 (61.8)	108 (60.3)	0.757
White, non-Hispanic	341 (83.6)	197 (83.5)	144 (83.7)	0.947
Single	225 (53.8)	131 (54.6)	94 (52.8)	0.719
Completed high school	304 (72.6)	171 (71.0)	133 (74.7)	0.393
*Structural vulnerabilities*				
Homelessness	235 (56.0)	127 (52.7)	108 (60.3)	0.119
Uninsured	115 (27.4)	67 (27.8)	48 (26.8)	0.823
Unemployment	277 (66.0)	157 (65.2)	120 (67.0)	0.685
Food insecurity	272 (64.8)	145 (60.2)	127 (71.0)	0.022
Recent arrest^a^	141 (33.6)	70 (29.1)	71 (39.7)	0.023
*Injection drug use, past* 6 months				
Cocaine	144 (34.3)	63 (26.1)	81 (45.3)	< 0.001
Crystal methamphetamine	298 (71.1)	150 (62.5)	148 (82.7)	< 0.001
Speedball	161 (38.3)	69 (28.6)	92 (51.4)	< 0.001
Opioids				
Heroin	340 (81.0)	171 (71.0)	169 (94.4)	< 0.001
Fentanyl	230 (54.8)	97 (40.3)	133 (74.3)	< 0.001
Prescription opioids	99 (23.6)	46 (19.1)	53 (29.6)	0.012
Buprenorphine or suboxone	127 (30.2)	72 (29.9)	55 (30.7)	0.851
*Non-injection drug use, past* 6 months^†^				
Cocaine	251 (59.8)	123 (51.0)	128 (71.5)	< 0.001
Crystal methamphetamine	234 (55.7)	120 (49.8)	114 (63.7)	0.005
Opioids				
Heroin	141 (33.6)	65 (27.0)	76 (42.5)	0.001
Fentanyl	38 (9.1)	14 (5.8)	24 (13.4)	0.007
Prescription opioids	149 (35.5)	75 (31.1)	74 (41.3)	0.030
Buprenorphine or suboxone	122 (29.1)	64 (26.6)	58 (32.4)	0.192
Sedatives	112 (26.7)	45 (18.7)	67 (37.4)	< 0.001
Tranquilizers	143 (34.1)	62 (25.7)	81 (45.3)	< 0.001
Stimulants	52 (12.4)	23 (9.5)	29 (16.2)	0.041
Hallucinogens	40 (9.6)	11 (4.6)	29 (16.2)	< 0.001
*Substance use*				
Number of drug types injected (mean, SD)	3.3 (1.7)	2.8 (1.5)	4.1 (1.7)	< 0.001
Use drugs with other people	287 (68.3)	153 (63.5)	134 (74.9)	0.013
Use drugs in public/semi-public spaces	199 (48.9)	99 (42.1)	100 (58.1)	0.001
Witnessed an overdose^a^	305 (73.0)	146 (60.8)	159 (89.3)	< 0.001
Tried to quit drug Use^a^	313 (74.5)	163 (67.6)	150 (83.8)	< 0.001

^a^All variables marked with an asterisk indicate exposure in the past 6 months

There were significant differences (p < 0.05) between PWID who had and had not recently experienced a NFOD (Table 1). Compared to their counterparts who had not experienced a recent NFOD, a greater proportion of people who recently overdosed reported experiencing food insecurity (71.0% and 60.2%, respectively). We also found several differences between persons with and without recent histories of overdose in terms of substance use-related measures; for example, persons who recently overdosed reported having recently injected a greater number of drug types than their counterpart PWID (4.1 and 2.8, respectively). People who reported recent overdose also reported more injection use of several specific drugs than those who had no recent overdoses, including cocaine (45.3% and 26.1%, respectively), crystal methamphetamine (82.7% and 62.5%, respectively), heroin (94.4% and 71.0%, respectively), fentanyl (74.3% and 40.3%, respectively), and speedball (51.4% and 28.6%, respectively). A greater proportion of PWID who reported having recently overdosed indicated they used drugs in a public space than their counterparts who had not recently overdosed (58.1% and 42.1%, respectively). In addition, there were significant differences between PWID who had and had not recently overdosed in terms of having recently witnessed an overdose and tried to quit using drugs.

Variables included in the multivariate analysis included food insecurity, number of drugs injected, use of drugs in a public space, witnessing an overdose in the past 6 months, and trying to quit using drugs in the past 6 months. In the multivariate analysis, factors significantly associated with recent overdose were number of drugs injected (aPR = 1.16; 95% CI 1.10, 1.23), witnessing an overdose in the past 6 months (aPR = 2.28, 95% CI 1.48, 3.50), and having tried to quit using drugs in the past 6 months (aPR = 1.54, 95% CI 1.11, 2.14) (Table 2).

**Table 2:** Multivariate log binomial regression model for experiencing a recent nonfatal overdose among people who inject drugs in Cabell County, WV 2018 (*n* = 405)

	PR	CI	*P* value	aPR	CI	*P *value
Food insecurity	1.32	(1.03, 1.71)	0.028	1.07	(0.87, 1.31)	0.539
Number of drugs injected	1.24	(1.19, 1.30)	< 0.001	1.16*	**(1.10, 1.23)**	**< 0.001**
Use drugs in public space	1.45	(1.15, 1.83)	0.002	1.03	(0.82, 1.30)	0.787
Witnessed an overdose in past 6 months	3.01	(2.03, 4.74)	< 0.001	**2.28**	**(1.48, 3.50)**	**< 0.001**
Tried to quit drug use in past 6 months	1.77	(1.27,2.46)	0.001	**1.54**	**(1.11, 2.14)**	**0.009**

Bold values indicate *p* <.05

## Discussion

Among our sample of PWID in West Virginia, nearly 43% reported having experienced at least one recent overdose and, among this group, the average number of reported recent overdoses was more than five. The prevalence of overdose in this sample far exceeds the global average among PWID: A recent meta-analysis estimated that the prevalence of recent (past 12 months) overdose among PWID globally was 20.5% [7]. Our estimate also exceeds those from other rural Appalachian populations: a 2011 study conducted in Kentucky estimated a 28% lifetime prevalence of NFOD [24]. There are several possible reasons the prevalence in our sample exceeds the rates found in related studies. For example, our participants were characterized by a number of structural vulnerabilities, like homelessness, which are known correlates of NFOD [7]. Our results may also reflect the increasing prevalence of fentanyl in the illicit drug supply [41].

Nonfatal overdose was associated with: injecting a greater number of drugs, witnessing an overdose in the past 6 months, and attempting to quit using drugs in the past 6 months. Our findings suggest that polysubstance use may be an underlying driver of overdose among rural PWID. This finding is consistent with recent studies that demonstrate increased risk of overdose with polysubstance use [17, 42]. While we cannot ascertain that persons in our sample were co-using multiple drugs, the higher counts of drugs recently injected parallels national data which demonstrates increasing rates of co-use, such as fentanyl and stimulants [43]. The high prevalence of polysubstance use among our rural sample of PWID illustrates the need for tailored overdose prevention initiatives, such as those that employ evidence-based harm reduction strategies aimed at reducing overdose risks among persons who use multiple classes of drugs (e.g., drug checking). Future work is needed to better understand polysubstance use in rural communities across the USA as well as the diversity of factors driving overdose in these communities.

Experiencing a recent NFOD was significantly associated with witnessing an overdose in the past 6 months. Furthermore, the prevalence of witnessing an overdose in the past 6 months in our sample was high (73.0%). This finding is consistent with national and international studies reporting that substantive proportions of PWID have witnessed an overdose: a recent systematic review found that the proportion of PWID reporting recently (past 6 or 12 months) witnessing an overdose ranged from 22 to 84% [7]. Both rural and urban studies in the USA have identified witnessing an overdose as a correlate of experiencing a NFOD [17, 24]. These findings emphasize the importance of centering PWID in overdose prevention efforts. For example, training PWID in naloxone administration and ensuring that they have a sufficient supply of overdose prevention resources (e.g., naloxone and fentanyl test strips) are important strategies to reduce overdose rates.

This is one of the first studies conducted among PWID in Appalachia to quantitatively link experiencing a NFOD with attempting to quit using drugs. Because our data are cross-sectional, we are not able to characterize the temporal relationship between these variables. However, experiencing an overdose may increase a person’s motivation to quit using drugs [18]. Another potential explanation for this association is that PWID who attempt to quit using drugs may lose their physiologic tolerance for opioids. If people with decreased tolerance for opioids return to substance use, they are at increased risk of overdose, especially if potent opioids such as fentanyl are prevalent in the drug supply. Regardless of the reasons driving the association between PWID attempting to quit using drugs and recent NFOD, it remains notable that a large proportion of rural PWID reported attempting to change their substance use behaviors. Longitudinal studies among PWID in rural Appalachia should further explore the relationship between NFOD and attempts to quit using drugs as well as enhance our understanding of the factors that support sustained substance use cessation.

In recent years, there have been signs of progress in WV regarding the reduction of overdose fatalities. Data show that the opioid overdose fatality rate decreased 14.5% between 2017 and 2018 at the state level [3]. However, multiple barriers to overdose prevention persist across the state. For example, stigma against people who use drugs has led to “not in my backyard” (NIMBY) opposition to harm reduction programs, which are crucial for access to naloxone and referrals to substance use treatment programs [29]. Additionally, access to evidence-based drug treatment options in WV is insufficient. A recent analysis demonstrated that less than 10% of Medicaid recipients received MOUD or mental health counseling after a NFOD, lower than rates reported in other states [44].

Given the significant volume of overdose fatalities in rural areas and the high prevalence of both experiencing and witnessing an overdose, future policy interventions should scale up access to overdose risk reduction tools for PWID in WV, including naloxone and fentanyl test strips [45]. Several promising policy options may increase access to these needed overdose prevention tools. For example, access to harm reduction programs in rural settings often lags behind urban counterparts, and clients may have to travel long distances to access services [25]. Expanding harm reduction sites and implementing mobile harm reduction services may be an effective way to reach high-risk groups of PWID [45, 46]. Beyond mobile harm reduction services, rural communities may also consider implementing public health vending machines in which persons may access supplies (e.g., naloxone and safe sex kits) to mitigate overdose and infectious disease acquisition risks [47–50].

Increasing access to MOUD for people who have experienced NFOD is another strategy that decreases fatal and nonfatal overdose. A recent spatial analysis from Vancouver, Canada, demonstrated that a greater availability of methadone clinics was associated with decreased odds of persons living in an overdose cluster [51]. Access to MOUD may have harm reduction potential even outside of formal treatment; for example, a recent study demonstrated that greater frequency of non-prescribed buprenorphine use was associated with decreased risk of overdose [52]. Additional promising strategies to reduce overdose fatalities include innovation in treatment program operations, such as: lowering the threshold to access treatment by eliminating the requirement to maintain abstinence from drugs, developing mobile programs that offer treatment to marginalized populations, or offering treatment at harm reduction programs [53–55]. In addition, expanding harm reduction programs throughout rural communities may also enhance overdose prevention via referrals to drug treatment programs and provision of overdose prevention kits. As noted by the Centers for Disease Control and Prevention, “…new users of [syringe services programs] are five times more likely to enter drug treatment and three times more likely to stop using drugs than those who don’t use the programs” [56].

This analysis has limitations to consider. It is plausible that our outcome measure did not capture overdose experiences that involved less severe physiologic changes (e.g., skin discoloration). We also did not have information on psychiatric comorbidities, which have been linked to NFOD in the literature [24]. We recorded counts of substances used in the past 6 months, but did not explicitly ask about simultaneous co-use. Exploring simultaneous co-use of multiple substances is an important realm of future inquiry as specific drug combinations may be strong drivers of overdose (e.g., sedatives/tranquilizers concurrently used with opioids). Some degree of survival bias may be present in our sample as we only examined correlates of NFOD. The results from our study may not be widely generalizable as our sample was drawn from a single county in WV with little sociodemographic diversity.

In conclusion, our research illustrates that NFOD among our sample of PWID in rural Appalachia was associated with injecting a greater number of drug types, witnessing an overdose in the past 6 months, and attempting to quit using drugs in the past 6 months. These findings reinforce results from urban studies that link polysubstance use and witnessing an overdose to experiencing a NFOD and build on those findings by linking NFOD to attempting to quit using drugs. This study underscores the urgency of increasing access to evidence-based overdose prevention resources, including harm reduction services, throughout rural America. Future research should investigate longitudinal shifts in overdose risks among rural PWID.

## Data Availability

The datasets generated and/or analyzed during the current study are not publicly available due to concerns about confidentiality.

## Abbreviations

PWID: People who inject drugs
US: United States of America
WV: West Virginia
NFOD: Nonfatal overdose
MOUD: Medications for opioid use disorder
